# Radiation emergency medical countermeasures: Current formulary, identified gaps, and future approaches

**DOI:** 10.1002/ame2.70109

**Published:** 2025-11-13

**Authors:** Wen‐Bing Ma, Rong‐Hua Yin, Guang‐Ming Ren, Yin Zhang, Li‐Juan Li, Wei Liu, Ting Chen, Wei Zhou, Mian Zu, Rong‐Ling Yin, Xin‐Di Shi, Lei Wang, Xiao‐Ming Yang

**Affiliations:** ^1^ Academy of Military Medical Sciences Beijing China; ^2^ State Key Laboratory of Medical Proteomics, Beijing Proteome Research Center, National Center for Protein Sciences (Beijing) Beijing Institute of Radiation Medicine Beijing China

**Keywords:** acute radiation syndrome, ionizing radiation, radiation injury, radiation medical countermeasures

## Abstract

Radiological or nuclear accidents can lead to serious outcomes for individuals exposed to ionizing radiation, with health effects that are either acute or delayed, deterministic or stochastic, depending on the effective dose of exposure. Mechanistically, ionizing radiation can inflict damage either directly on DNA or through oxidative stress, which may trigger a cascade of damages to tissues and organs. The development of effective radiation medical countermeasures is an unmet need and should be a top priority in preparing for radiation emergencies. This paper aims to address the critical questions of whether current countermeasures are available, what additional measures are needed, and what actions can be taken to enhance the development of radiation medical countermeasures from a systematic perspective.

## Graphical abstract

Current radiation medical countermeasures primarily focus on hematopoietic acute radiation syndrome (H‐ARS) using agents like granulocyte colony‐stimulating factor (G‐CSF), granulocyte‐macrophage colony‐stimulating factor (GM‐CSF), and romiplostim, alongside treatments for internal contamination (Prussian blue, diethylenetriamine pentaacetate [DTPA]). Critical gaps persist, including a lack of prophylactic agents, narrow therapeutic time windows, and insufficient therapies for gastrointestinal, cardiovascular, and other organ‐specific effects. Promising emerging approaches—such as drug repurposing, novel targeted agents (e.g., mitochondrial antioxidants), stem cell therapies, and combinatorial regimens—hold potential to fill these gaps. Their development is supported by advanced animal models and optimized regulatory guidelines to accelerate clinical translation.

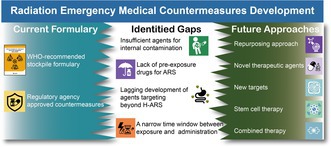

## 1 INTRODUCTION

Radiological and nuclear threats may occur at facilities or in transportation vehicles containing radioactive materials, or come from radiological and nuclear weapons or devices. Whether intended or unintended, natural or man‐made, the outcomes of a radiological or nuclear incident can be catastrophic and may impose serious radiation injuries on individuals after exposure. Tissue damage induced by ionizing radiation (IR) may be acute, subacute, or delayed. One of the most serious health outcomes is acute radiation syndrome (ARS), which is a systemic effect after a high dose and high‐dose rate exposure, manifesting as hematopoietic (H), gastrointestinal (GI), neurovascular subsyndromes. Notably, as historical incident data reveal, radiation combined injuries (RCIs), which occur when radiation injury is coupled with other forms of injury, such as burns, wounds, trauma, or sepsis, can be common injuries in some emergency accidents.^1^ RCIs are manifested as shock, severe gastrointestinal (GI) damage, dramatic suppression of hematopoietic and immune systems, and delayed wound healing.^2^ The concurrent immune system impairment significantly increases susceptibility to life‐threatening infections.^3^ After surviving the acute phase, individuals may experience some delayed effects of acute radiation exposure (DEARE), with evolving pathologies of multiple organ systems and serious outcomes, such as multiple organ failure or death, when these pathological syndromes are fully expressed.^4^ Additionally, during the acute phase of a radiation emergency, some individuals who have been exposed may develop cutaneous radiation injury (CRI). This condition can present with initial symptoms such as itchiness, tingling sensations, and skin redness, among others. Exposure to radiation can also lead to some chronic and stochastic health effects, with cancer being a notable example. The likelihood of developing such conditions is dose‐dependent, and higher doses increase the probability and/or severity of these adverse health outcomes. These long‐term effects can occur in exposed individuals or offsprings due to prenatal exposure, whereas some effects may be transgenerational due to paternal DNA damage.^5^

The mechanisms behind the health effects of radiation are intricate and often unfold in a cascade pattern. The primary early deleterious impact is caused by DNA damage and oxidative stress. Cellular DNA is highly susceptible to ionizing radiation, with sublethal doses leading to multiple DNA lesions.^6^ Despite the sophisticated repair mechanisms within the nucleus, some damage remains unrepaired or is misrepaired, leading to cellular dysfunction and possible cell death. In addition to the direct deposition of energy, the damage can arise from free radicals produced by the ionizing effects on water, which account for 70% of injuries induced by IR.^6^ The cell damage imposed by DNA damage and reactive oxygen species (ROS) can trigger a pro‐inflammatory reaction through the release of damage‐associated molecular patterns (DAMPs),^7^ which may lead to excessive immune response and exacerbate the radiation‐induced tissue or organ damage. Notably, besides DNA and DAMPs, radiation can inflict damage to other cellular compartments, with evidence revealing that endothelial acid sphingomyelinase is activated after radiation exposure, catalyzing ceramide generation on the plasma membranes of mammals to initiate apoptotic signaling.^8^, ^9^, ^10^

Radiation emergency preparedness represents a long‐standing goal for many countries, which is severely hindered by the limited availability of radiation medical countermeasures (MCMs). This article reviews both clinically approved agents and promising candidates under development, with an emphasis on analyzing the direction we should pursue and how emerging therapeutics, protocols, and drugs might drive the evolution of future standard therapy formulations.

Radiation emergency preparedness is a critical aspect of national security, encompassing a wide range of activities, including guideline development, stockpile management, and the establishment of response teams. Among these activities, a key element is the development of radiation MCMs, which is a challenging and complex process, as there are now only a limited number of proven and reliable MCMs available. As a result, there remains a need to formulate a detailed landscape of the current status, which may guide future efforts.

## 2 CURRENT RADIATION MEDICAL COUNTERMEASURES

### 2.1 The World Health Organization‐recommended stockpile formulary

On January 23, 2023, the World Health Organization (WHO) published a report on national stockpiles for radiological and nuclear emergencies.^11^ This report provides policy recommendations at the national level for handling radiological and nuclear emergencies, with an emphasis on national stockpile establishment and management. Based on advances in radiation emergency medicine over the past dozen years, the report updates a 2007 list of medicines for use in radiation and nuclear emergencies, which recommends several generic drugs such as Prussian blue, calcium diethylenetriamine pentaacetate (Ca‐DTPA), zinc diethylenetriamine pentaacetate (Zn‐DTPA), and granulocyte colony‐stimulating factor (G‐CSF).

The updated list mainly focuses on the prevention of internal contamination and ARS. For internal contamination, a blocking agent (potassium iodide) and several decorporating agents are covered, which include Prussian blue, Ca‐DTPA or Zn‐DTPA, aluminum‐containing antacids and alginates, and sodium bicarbonate. ARS can manifest as hematopoietic, GI, and neurovascular subsyndromes. The WHO report only focuses on the first two types due to the fact that neurovascular subsyndrome is nonsalvageable (Table 1).

**TABLE 1:** WHO stockpile formulary for acute radiation syndrome (ARS).

Agents	Drug name	Route of application
*Agents for hematopoietic subsyndromes*		
Granulocyte colony‐stimulating factor (G‐CSF)	Filgrastim (no glycosylation), lenograstim (with glycosylation)	Subcutaneous injection
Pegylated granulocyte colony‐stimulating factor (PEG‐G‐CSF)	Pegfilgrastim	Subcutaneous injection
Granulocyte‐macrophage colony‐stimulating factor (GM‐CSF)	Sargramostim	Subcutaneous injection
Thrombopoietin receptor agonist	Romiplostim	Subcutaneous injection
*Agents for GI subsyndromes*		
Antiemetic	Ondansetron	Intravenous infusion, orally as melting tablets or film tablets
Antidiarrheal	Loperamide	Oral

### 2.2 US Food and Drug Administration‐approved countermeasures

Currently, the US Food and Drug Administration (FDA) has approved 16 agents for radiation MCMs. Of these, 11 target the hematopoietic syndrome of acute radiation syndrome (H‐ARS) (Table 2), 1 (Silverlon) addresses cutaneous radiation injuries, and the remaining 4 are designed to eliminate internal radioactive contamination, including Prussian blue (radiogardase), potassium iodide, Ca‐DTPA, and Zn‐DTPA.

**TABLE 2:** US FDA‐approved radiation medical countermeasures for H‐ARS.

Agents	Manufacturer	Initial US approval/for H‐ARS indication	Mechanism of action	Dosage form and administration route	Condition of usage	Animal models
Neupogen (filgrastim)	Amgen Inc.	1991/2015.03	Promotes the growth of neutrophils, thus alleviating the neutropenia and preventing infection	Vial solution and prefilled syringe, for injection	Administer Neupogen as soon as possible after suspected or confirmed exposure to radiation doses greater than 2 gray (Gy)	Canine,^12^, ^13^ mice,^14^, ^15^, ^16^, ^17^, ^18^ minipig,^19^ rhesus macaques^20^
Neulasta (pegfilgrastim)	Amgen Inc.	2002/2015.11	Promotes the growth of neutrophils, thus alleviating the neutropenia and preventing infection	Solution, for injection	Administer the first dose as soon as possible after suspected or confirmed exposure to radiation levels greater than 2 gray (Gy). Administer the second dose 1 week after the first dose	Mice,^21^, ^22^ rhesus macaques,^23^, ^24^ minipig,^25^ rat^22^
Leukine (sargramostim)	Sanofi‐Aventis U.S. LLC	1991/2018.03	Stimulates the growth of certain white blood cells that are important in the body's fight against infection	Lyophilized powder and solution, for injection	Administer Leukine as soon as possible after suspected or confirmed exposure to radiation doses greater than 2 gray (Gy)	Canine,^26^ mice,^27^ rhesus macaques^28^
Nplate (romiplostim)	Amgen Inc.	2008/2021.01	Increases platelet production through binding and activation of the thrombopoietin (TPO) receptor, thus alleviating the thrombocytopenia and preventing bleeding	A lyophilized powder in single‐dose vials, injection	Administer the dose as soon as possible after suspected or confirmed exposure to radiation levels greater than 2 gray (Gy)	Mice,^29^ rhesus macaques^30^
Udenyca (pegfilgrastim‐cbqv), biosimilar to Neulasta	Coherus BioSciences, Inc.	2018/2022.11;2023.11 (single‐dose prefilled auto‐injector presentation)	Promotes the growth of neutrophils, thus alleviating the neutropenia and preventing infection	Prefilled syringe for manual use only; prefilled auto‐injector	Administer the first dose as soon as possible after suspected or confirmed exposure to radiation levels greater than 2 gray (Gy). Administer the second dose 1 week after the first dose	Rhesus macaques (similar to Neulasta as disclosed by product labeling information)
Stimufend (pegfilgrastim‐fpgk), biosimilar to Neulasta	Fresenius Kabi USA, LLC	2022/2023.09	Promotes the growth of neutrophils, thus alleviating the neutropenia and preventing infection	Prefilled single‐dose syringe for manual use	Administer the first dose as soon as possible after suspected or confirmed exposure to radiation levels greater than 2 gray (Gy). Administer the second dose 1 week after the first dose	Rhesus macaques (similar to Neulasta as disclosed by product labeling information)
Ziextenzo (pegfilgrastim‐bmez), biosimilar to Neulasta	Sandoz Inc.	2019/2024.02	Promotes the growth of neutrophils, thus alleviating the neutropenia and preventing infection	Single‐dose prefilled syringe	Administer the first dose as soon as possible after suspected or confirmed exposure to radiation levels greater than 2 gray (Gy). Administer the second dose 1 week after the first dose	Rhesus macaques (similar to Neulasta as disclosed by product labeling information)
Nypozi (filgrastim‐txid), biosimilar to Neupogen	Tanvex BioPharma USA, Inc.	2024.06	Promotes the growth of neutrophils, thus alleviating the neutropenia and preventing infection	Injection with a prefilled syringe	Administer Nypozi as soon as possible after suspected or confirmed exposure to radiation doses greater than 2 gray (Gy)	Rhesus macaques (similar to Neupogen as disclosed by product labeling information)
Zarxio (filgrastim‐sndz), biosimilar to Neupogen	Sandoz Inc.	2015/2024.10	Promotes the growth of neutrophils, thus alleviating the neutropenia and preventing infection	Vial solution and prefilled syringe, for injection	Administer Zarxio as soon as possible after suspected or confirmed exposure to radiation doses greater than 2 gray (Gy)	Rhesus macaques (similar to Neupogen as disclosed by product labeling information)
Fylnetra (pegfilgrastim‐pbbk), biosimilar to Neulasta	Kashiv BioSciences, LLC	2022/2025.04	Promotes the growth of neutrophils, thus alleviating the neutropenia and preventing infection	Prefilled single‐dose syringe for manual use	Administer the first dose as soon as possible after suspected or confirmed exposure to radiation levels greater than 2 gray (Gy). Administer the second dose 1 week after the first dose	Rhesus macaques (similar to Neulasta as disclosed by product labeling information)
Releuko (filgrastim‐ayow), biosimilar to Neupogen	Kashiv BioSciences, LLC	2022/2025.04	Promotes the growth of neutrophils, thus alleviating the neutropenia and preventing infection	Vial solution and prefilled syringe, for injection	Administer Releuko as soon as possible after suspected or confirmed exposure to radiation doses greater than 2 gray (Gy)	Rhesus macaques (similar to Neupogen as disclosed by product labeling information)

## 3 CURRENT GAPS IN RADIATION MCMS DEVELOPMENT

### 3.1 Insufficient agents for internal contamination

The WHO has recommended several agents for internal contamination, but as stated in its reports, these cover only a narrow range of radionuclides, and some have limitations for usage, especially in mass casualty emergencies. For example, Ca‐DTPA and Zn‐DTPA are used for decorporation of transuranium elements and rare earth radionuclides but are not recommended for decorporation of U, Np, or Cd due to potential nephrotoxicity. Moreover, although intravenous injection remains the primary recommended route for DTPA administration, simpler alternatives—particularly oral delivery—are needed under mass casualty scenarios. However, low intestinal permeability and limited bioavailability of current oral Ca/Zn‐DTPA formulations severely compromise their efficacy, rendering them unsuitable for emergency use. This necessitates urgent development of optimized delivery strategies,^31^ with ongoing efforts reported, such as improving the oral bioavailability^32^, ^33^ of Ca/Zn‐DTPA or other substitute chelators.^34^ The major limitations of Prussian blue are its efficiency constraints. Its decorporation efficiency for cesium (Cs) is compromised when treatment initiation is delayed and is inherently limited by the crystal structure of Prussian blue, which restricts its Cs adsorption rate.^35^ Furthermore, concerns exist regarding side effects, including hypokalemia, GI damage, and constipation.^36^ Several attempts have been made to improve the decorporation efficiency of Prussian blue through preparation in the form of nanoparticles,^37^, ^38^ Prussian blue analogs, or composite materials.^39^ The primary disadvantage of KI is that it protects only the thyroid and has a relatively narrow time window for maximum effectiveness,^40^ with the recommended optimal time of administration by WHO to be within 24 h before and 2 h after exposure.^11^ These facts call for improved formulation or novel agents to improve bioavailability or efficiency.

### 3.2 Lack of pre‐exposure drugs for ARS

Radiation MCMs can be categorized into three types based on the timing of their administration: radioprotectors, radiomitigators, and radiation therapeutics. Radioprotectors should be administered prior to IR exposure to prevent severe radiation injuries, whereas radiomitigators are intended to be administered shortly after IR exposure to mitigate and promote recovery from radiation injuries. Radiation therapeutics are used when the symptoms of radiation have fully manifested. Currently, only a limited number of countermeasures for ARS have been officially approved or recommended, and all of these are recommended to be administered after IR exposure. We lack effective prophylaxis agents with minimal side effects for ARS, which can help build the first line of medical defense against a radiation emergency accident and will be invaluable for soldiers, first responders, or civilians during evacuation, where they face the risk of secondary exposure from successive nuclear explosions. There are some leading chemical or natural radioprotectors under development, such as Ex‐RAD,^41^ gamma‐tocotrienol,^42^ amifostine,^43^ BIO 300,^44^ DMSO,^45^ TLR5 agonist,^46^ and so on.^47^, ^48^ Recent advances in applying ester prodrug strategies have further enhanced the potential of tocotrienols as radioprotectors.^49^ Cao et al. fabricated a PEGylated MIL‐101(Fe) nanoparticle drug carrier to codeliver WR‐1065 (the active metabolite of amifostine) and glutathione (GSH) to optimize the oral activity and decrease the cytotoxicity.^50^ If successfully developed, these agents can be incorporated into national stockpiles for emergencies. In parallel to pharmacological developments, certain foods and nutraceuticals have demonstrated radioprotective properties that may serve as accessible initial interventions during early‐phase exposure.^51^, ^52^, ^53^, ^54^, ^55^ Although not equivalent to targeted drugs, these substances can be deployed rapidly on a large scale due to their favorable logistics, nutritional value, and lack of clinical contraindications.

### 3.3 Lagging development of agents targeting beyond H‐ARS

Currently approved countermeasures are mainly centered on H‐ARS, especially neutropenia or thrombocytopenia symptoms. The development of agents targeting other symptoms, subsyndromes, or DEARE for treating radiation injuries of GI, cardiovascular, neurological, lung, kidney, or other system compartments of the body is slow in progress. This is especially true for GI‐ARS; though the WHO's updated report has recommended ondansetron and loperamide for GI symptom relief, these two agents are supportive therapies aimed at controlling nausea, vomiting, and diarrhea. Developing countermeasures that limit further tissue damage or promote recovery represents a more accurate and efficient way for controlling GI‐ARS. Currently, the number of repurposing candidates for this aim is limited, and the situation calls for innovative therapies and new targets, which are based on better characterization of the injuries of GI‐ARS across molecular, cellular, tissue, and microbiome landscapes, as well as the development of animal models to better simulate human responses.^56^

### 3.4 A narrow time window between exposure and administration

The currently approved agents for H‐ARS are all indicated for administration as soon as possible after radiation exposure, presumably due to reduced efficacy outside the optimal administration window.^57^ As illustrated by Neupogen, the improvement in survival is not statistically significant when administered 48 h post‐total body irradiation (TBI) in rhesus macaques,^58^ whereas a significant improvement is observed when administered within 24 h postexposure,^20^ suggesting a rigid interval‐dependent timing between irradiation and administration. The time window is important, because in real‐world circumstances, some individuals may not receive timely treatment due to inadequate triage or drug delivery logistics, as demonstrated by the human experience of past accidents.^59^ Early diagnosis using lymphocyte depletion kinetics is recommended, which is critical for initiating treatment.^60^, ^61^ In 2023, the US National Institute of Allergy and Infectious Diseases (NIAID), National Institutes of Health (NIH), issued a funding opportunity for the development of radiation/nuclear MCMs, with a particular interest in MCMs that retain efficacy beyond 24 h.^62^ Some ongoing development has shown promising results. For example, captopril, an angiotensin‐converting enzyme (ACE) inhibitor, retains efficacy as late as 48 h postradiation in mice.^63^ Som230, a somatostatin analog, shows significant survival benefits for GI‐ARS when administered at 72 h postirradiation.^64^ It should also be noted that evidence shows that sargramostim still retains some survival and hematological benefits when the administration is initiated up to 96 and 120 h postexposure, respectively.^65^

## 4 PROMISING APPROACHES OR CANDIDATES

### 4.1 Repurposing approach

As indicated by the approval history, a number of approved agents for H‐ARS are repurposed from past usage.^57^, ^66^ The repurposing approach is a time‐ and cost‐efficient path for drug development. This concept is particularly true for radiation protection drug development, as no human cohorts are appropriate for clinical efficacy examination, which is usually based on the US FDA Animal Rule (21 CFR 314 subpart I for drugs and 21 CFR 601 subpart H for biological products), whereas the safety considerations can be built on past clinical studies in patients with other approved indications. The updated WHO report has indeed recommended investigational therapies that may be repurposed, such as lung surfactants and ACE inhibitors originally for premature infants at risk of neonatal respiratory distress syndrome and for treating hypertension and heart failure, respectively. In fact, there is a plethora of agents that have repurposing potential for radiation emergency use, including some drugs used in cancer radiotherapy and for other indications.^66^, ^67^

### 4.2 Novel countermeasures development

#### 4.2.1 Novel therapeutic agents

Promising radiation MCMs are expanding, including potential protective, mitigative, and therapeutic agents,^40^, ^68^, ^69^, ^70^ and some have entered into advanced development.^71^ CBLB502, a TLR5 agonist, was originally designed to mimic cancer's antiapoptotic mechanisms, aiming to endow cells with radioprotective effects. The inventors adopted an approach of utilizing bacterial protein flagellin to stimulate NF‐κB, thereby inhibiting the p53 pathway.^72^ The resulting drug was found to be an effective countermeasure against ARS in animal models and has now been granted by the FDA investigational new drug and orphan drug status.^73^ Another p53 signaling pathway‐targeted agent,^74^ known as EX‐RAD, has been shown to be involved in the PI3‐kinase/AKT pathway.^75^ It exerts protective effects on the hematopoietic and GI systems by safeguarding cells from DNA damage, inhibiting apoptosis and promoting cell survival.^76^ BBT‐059, a pegylated construct of interleukin 11 (IL‐11), is a promising radiation MCM with an expanded time window for treating H‐ARS.^77^ BIO 300, which is under development for H‐ARS and DEARE, has reported positive phase I results (BIO 300 Oral Powder Safety and Pharmacokinetics; NCT04650555)^78^ and is being investigated following the FDA Animal Rule pathway.^79^, ^80^ Similarly, KMRC011, a new toll‐like receptor 5 agonist, has been investigated in a human study of tolerability, pharmacokinetics, and pharmacodynamics as a treatment for H‐ARS.^81^ Wang et al. have demonstrated that the small molecule Me6TREN stimulates intestinal stem cell proliferation, thereby promoting regeneration of radiation‐injured intestinal tissue.^82^ Some Chinese herbal medicines have also been investigated for the protective or mitigative effects of intestinal radiation damage.^83^, ^84^, ^85^ The search for potential countermeasures is expanding,^86^, ^87^ and a detailed enumeration of these advancements is outside of the scope of this paper.

#### 4.2.2 New targets for MCM development

There have been reviews of potential targets for radiation MCMs development, such as DNA damage, ROS/nitric oxide (NO) generation, and cell death.^70^ These signaling pathways are instructive for drug development through a target‐based drug discovery (TBDD) approach. Most currently approved or candidate radiation MCMs have been developed through the phenotype‐based drug discovery (PBDD) method,^88^ which is either initiated through phenotype‐related repurposing attempts or through phenotypic high‐throughput assays. However, neither TBDD nor PBDD approaches provide a one‐size‐fit‐all solution for radiation MCMs development because of the complexity of radiation‐induced injuries leading to multiorgan involved (MOI) injury or multiorgan failure (MOF). This well‐known radiation exposure complication underscores the importance of better characterization of disease mechanisms occurring in different organs under different qualities of radiation. As illustrated by the development of the anticeramide antibody against GI‐ARS, endothelial cells were identified as the primary cell damage compartment,^89^ and radiation‐induced changes to ceramide‐initiated apoptosis in the endothelium are noteworthy. The resultant anticeramide single‐chain variable fragment (scFv) has demonstrated its potential to serve as both a therapeutic and a prophylactic measure against GI‐ARS.^9^, ^10^

Mitochondria are the primary sources of physiological ROS and possess important antioxidant defenses. These antioxidant defenses can be utilized or modified during radiation‐induced injury. Yang et al. devised mitochondrial‐targeted cerium oxide nanoclusters,^90^ which can stimulate superoxide dismutase and catalase activities, and have indicated that they exert protective effects by targeting the mitochondria of extramedullary hematopoietic organs, especially liver and spleen, through continuously clearing ROS. Brand et al. have shown that JP4‐039, a gramicidin S‐conjugated cycling nitroxide, can act as a mitochondrial membrane‐targeted antioxidant through potent electron scavenging and thus reduce the clinical signs of skin damage in both mice and humans when applied pre‐ or postirradiation.^91^

Another potential new target compartment is the microbiome. Though direct evidence from human microbiome studies following radiation/nuclear accidents remains limited, many cancer radiotherapies and animal model‐based research have suggested that the microbiome influences the outcomes of radiation‐induced disease.^92^, ^93^, ^94^ The manipulation of human microbiome represents a promising novel intervention strategy for injuries induced by radiation. Approaches such as antibiotics, probiotics, dietary modification, and fecal microbiota transplantation may affect patient outcomes, whereas changes in the microbiome can be used as biomarkers to inform disease progression and treatment effects.^95^ Guo et al.^96^ have found that certain bacteria are highly enriched in both “elite‐survivors” that represent a small population of mice surviving from a high dose of radiation, and also in leukemia patients who received radiotherapy but suffer from mild adverse effects. Administration of certain metabolites that are selectively increased in the feces of these elite‐survivors provides protection to both hematopoietic and GI tissues. In 2021, the NIAID, NIH, posted a funding opportunity for the development of microbiome‐focused strategies for diagnosis, mitigation, or treatment of radiation injuries.^97^ As a result, award recipients have been working on creating innovative therapeutic agents targeting the microbiome.

#### 4.2.3 Stem cell therapy

Stem cell therapy is a highly promising treatment option for H‐ARS. Hematopoietic stem cells (HSCs) and mesenchymal stromal cells (MSCs) are two primary types of stem cells that have shown potential benefits in preclinical studies and clinical observations for mitigating the effects of ARS. Administration of allogeneic or unexposed autologous HSCs promotes the recovery of hematopoietic tissues, and there is also evidence suggesting that transplanted HSCs can regenerate nonhematopoietic tissues, such as myocardium and nerves. MSCs can secrete many cytokines, chemokines, and growth factors, which can promote hematopoiesis, tissue repair, and the maintenance of homeostasis.^98^, ^99^ There is also evidence supporting that MSCs possess antioxidant properties, which may at least be partly accountable for their cytoprotective and anti‐inflammatory effects.^100^ Accumulating evidence supports the use of MSCs in radiation scenarios, including hematopoietic, GI, and cutaneous ARS. It should be noted that PLacenta eXpanded‐R18 (PLX‐R18), MSC‐like cells that promote hematopoietic system recovery in mice following radiation exposure,^101^ is being tested in an ongoing phase I study. More supportive preclinical and clinical evidence surrounding its safety and efficacy is needed before regulatory agencies will approve its use to make them more suitable for radiation emergency usage, such as the development of a ready‐to‐use stem cell bank, which allows them to be more available in the face of sudden surges of cases.^102^

### 4.3 Combined therapy (polypharmacy)

From the point of clinical management of acute radiation sickness, high‐dose acute radiation exposure often results in injuries involving whole‐body, multiorgan systemic effects; therefore, treatment schema may involve multiple approaches, such as the administration of antibiotics, antiemetic compounds, antiviral agents.^103^ For therapy development, two or more composite formulas can either give rise to synergistic or additive effects compared to a single formula prescription. Amifostine is a powerful radioprotective agent but is approved only for limited clinical use in radiotherapy‐treated ovarian cancer and chemotherapy‐treated head and neck cancers. The main problem limiting its use in an ARS context is high toxicity associated with optimal dosing. One promising strategy is the polypharmacy approach, which can boost the protective effects of amifostine. Such attempts to test amifostine have been studied in multiple systems.^43^

Besides the combination of two or more countermeasures, the novel stem cell therapy can be modified. Chen et al. combined mesenchymal stromal cells with a genetic modification method to alleviate acute radiation‐induced lung injury, with the aim to constitutively express an antioxidant enzyme, namely manganese superoxide dismutase (MnSOD) that is localized in mitochondria.^104^ Similarly, Kim et al.^105^ adopted a combined method of bone marrow transplantation to attenuate GI‐ARS induced by radiation exposure. The agent used in combination was fibroblast growth factor 2, which has been implicated in protecting crypt stem cells from apoptosis and enhancing their survival after irradiation.^106^

## 5 MODELS FOR DRUG DEVELOPMENT

### 5.1 The FDA Animal Rule

Developing animal models for products investigated under the FDA Animal Rule is a critical process, as the models should be both demonstrative of the clinical conditions being targeted and reactive to the product being tested. Data from more than one animal species are usually required unless a single species is well characterized for predicting the human response as judged by the review agency. This process can be difficult, as illustrated by the case of Eltrombopag, which is an FDA‐approved agonist of the TPO receptor with indications for chronic hepatitis C patient thrombocytopenia, immune thrombocytopenia, and patients with aplastic anemia. Eltrombopag has a narrow selection of animal models, as it is only effective in chimpanzees and humans, which is due to the species specificity of the binding target.^57^ Chimpanzee testing is prohibited by the US NIH due to its endangered species status.

### 5.2 Animal model development

To study radiation injuries and develop MCMs, various species such as rodents, canines, rabbits, swine, and nonhuman primates (NHPs) have been employed. Rodents (mice and rats) have been used for H‐ARS,^107^ GI‐ARS,^108^ DEARE,^109^, ^110^ skin injury,^111^ and RCIs modeling.^112^ Their advantages include ease of handling, cost‐effectiveness, and relatively short lifespans suitable for long‐term or age‐related studies.^113^ However, their limitations include limited translatability of heterogeneous radiation dose distributions in humans due to small body thickness, minimal blood volume restricting sequential sampling, and absence of clear prodromal manifestations.^114^ Rabbits share rodents' advantages, such as ease of handling and cost‐effectiveness, while providing additional benefits: feasibility for sequential blood sampling and a human‐like coagulation profile, making them valuable for radiation‐induced coagulopathy research.^115^ Swine, such as Yorkshire and minipigs, share similarities in skin structure to human, making them suitable for modeling cutaneous radiation injuries,^116^, ^117^ whereas they have also been used for H‐ARS,^118^, ^119^, ^120^ GI‐ARS,^121^, ^122^ and RCIs research.^123^ While developing models for ARS, it should be noted that pigs might present higher sensitivity than humans, presumably due to the propensity to develop radiation‐induced disseminated intravascular coagulation,^124^, ^125^ and they are less prone to vomiting compared to canines and ferrets.^114^ Canines have been used for H‐ARS,^12^ GI‐ARS,^126^ and RCIs studies.^127^ They experience vomiting and diarrhea and possess similar hematopoietic and immune systems to humans, whereas the differences regarding pulmonary anatomy and function have been emphasized when considering the physiological differences between humans and canines.^128^ In addition, ethical concerns regarding their status as companion animals limit their desirability as a model species where alternative options exist. NHP models are considered the gold standard of animal models for ARS due to their genetic homology and tissue or organ similarity to humans. They have been used to model H‐ARS,^129^, ^130^, ^131^, ^132^ GI‐ARS,^133^, ^134^, ^135^ and DEARE^136^; however, NHPs are not used for skin injuries, as pig skin is closest to human skin.

Several exposure protocols have been established during the development of animal models, including unilateral or bilateral TBI, partial‐body irradiation with bone marrow sparing (PBI/BM sparing), and localized exposure (whole thorax lung irradiation [WTLI], total‐abdominal irradiation [TAI], and local skin irradiation). The appropriate model selection is related to the type of subsyndrome/injury and the incident scenario to be studied. For instance, the threshold dose for developing GI‐ARS is higher than H‐ARS; therefore, a PBI/BM‐sparing protocol enables the animal to survive the lethal phase of H‐ARS. During a nuclear terrorist incident or a detonation of a low‐yield nuclear weapon, uniform TBI might not mimic the real scenario.^137^ To address this concern, Accardi et al. have developed an oral cavity–shielding PBI protocol for testing orally administered MCMs.^135^ The WTLI model enables investigations focused on radiation‐induced lung injury, although the US FDA has indicated that PB/BM5 is preferred given its predictable evolution of lung‐DEARE and in the context of multiorgan injury.^138^ A US FDA draft guidance for developing drugs for ARS emphasizes that TBI, with or without partial BM sparing, is preferred for confirmatory studies compared to localized exposure protocols (e.g., WTLI or TAI).^139^

Another point surrounding the variety of animal model development is the fact that the medical response planners need to develop advanced planning, which incorporates population variabilities during preclinical and clinical studies, such as obstetric, pediatric, and geriatric populations and individuals with compromised immune systems.^140^ As for usage during pregnancy, currently approved treatments largely rely on animal data, which might indicate potential risks that need to be thoroughly balanced against the benefits, and the human data available are often inadequate to draw a definite conclusion.^141^ It should be noted that in a draft guidance issued by US FDA for developing drugs specific for ARS,^139^ the agency emphasizes that factors such as age and sex should be considered, and it is important to develop a pediatric study plan and determine whether sex‐based differences contribute to differential response of the treatment under investigation. This requirement underscores the importance of inclusion of more parameters when developing and characterizing the animal model used for radiation MCMs development.^142^, ^143^

### 5.3 Organ‐on‐chip models

Organ‐on‐chip models represent an emerging and paradigm‐shifting technology for drug development with the potential to replace steps in preclinical studies such as drug screening, disease modeling, and metabolism and pharmacokinetics analysis. As for radiation MCMs development, the organ‐on‐chip has the potential to reduce animal use and better model human systems, which is particularly critical for agents that have a narrow species specificity. There have been studies for radiation MCMs analysis using organ‐on‐chip models, including bone marrow–on–a–chip,^144^ gut‐on‐chip,^145^ alveolus‐on‐a‐chip,^146^ etc. Although organ‐on‐chip approaches are still in their infancy in terms of supporting regulatory approval, partly due to a lack of standardized models, advances in technology and regulatory guidance are needed. The US FDA has funded research aiming to develop lung, gut, and bone marrow organ‐on‐chips to test the effectiveness of radiation MCMs,^147^ and a parallel funding initiative was initiated by the US NIAID to develop alternative human models of radiation‐induced injuries.^148^

## 6 OTHER CONSIDERATIONS CLOSELY RELATED TO DRUG DEVELOPMENT

### 6.1 Guidelines

As emphasized by the updated WHO report, appropriate procedures for licensing and approval are required for managing national stockpiles for radiological or nuclear emergencies. This is the prerequisite not only for innovative therapies, such as stem cell therapy and biobanking, but also for common countermeasures development from the perspective of accelerating the development process. For development of ARS countermeasures, this pipeline can be more challenging as product development under the FDA Animal Rule can be more complex, which may involve more communication between sponsors and the review team, strict animal model selection, and high standard safety and efficacy considerations. Generally, the Animal Rule is adopted due to the ethical and operational infeasibility of human challenge studies and field trials. However, to some extent, as evidenced by US FDA recommendations and prior approval experiences with H‐ARS agents, the human clinical data accumulated in other conditions may be supportive for efficacy demonstration and integral for well‐characterized safety profile for use in radiation exposure–based scenarios.^149^

### 6.2 Stockpiling and triage

There are several therapeutics approved for radiation injuries, and we have accumulated abundant clinical management experiences; however, there may not be enough MCMs available especially when there are many victims of serious radiation incidents, which may disrupt the normal course of emergency and health services. The storage and logistics of MCMs are critical constraints in such scenarios, particularly for FDA‐approved biologics like filgrastim and romiplostim, which demand uninterrupted cold‐chain transport (2–8°C). Administration routes further dictate logistics: self‐administered oral agents (e.g., KI pills) enable community‐level distribution, whereas injectables require clinical settings and trained staff. Notably, prefilled syringes or solution formats for certain FDA‐approved MCMs (Table 2) expand flexibility by permitting home administration under a medical provider's instruction. To address these limitations, drug development efforts are actively exploring solutions, including agents like delta‐tocotrienol (delta‐T3H), which induces endogenous growth factors and shows promise as a temperature‐stable alternative that could circumvent cold‐chain requirements in future deployments.^150^ Approaches validated through mass COVID‐19 vaccination campaigns offer transferable insights for contingency planning, given operational parallels in cold‐chain dependency and the rapid surge in demand for time‐sensitive therapeutics.^151^, ^152^

Effective triage during radiation emergencies is critical not only for optimizing resource allocation by distinguishing patients requiring urgent intervention from those needing observation but also for guiding subsequent MCMs. As radiation injury‐specific therapeutics advance for complex injury patterns, future medical management must integrate triage with tailored treatment protocols. This imperative underscores the need for a viable clinical triage system during a radiation emergency, which calls for future innovative integration of dosimetry and automation tools for triage. In recent years, radiation dosimetry techniques have advanced toward rapid, high‐throughput, and precise methodologies. Automated testing platforms have been developed to overcome the time‐ and labor‐intensive constraints of traditional approaches.^153^, ^154^, ^155^, ^156^ Beyond conventional cytogenetic biomarkers (e.g., dicentrics, translocations, chromosomal fragments) and protein markers (e.g., 53BP1, γ‐H2AX), novel signatures, including new protein biomarkers,^157^ noncoding RNAs,^158^ and metabolites,^159^ are being actively explored. Multiomics strategies, such as proteomics,^160^ lipidomics,^161^ and metabolomics,^162^ further enable rapid, high‐throughput triage by comprehensively mapping radiation responses. Critically, machine learning frameworks integrating these multiomic biomarkers are enhancing biodosimetry accuracy and scalability.^163^ With numerous researchers focusing on deciphering biomarkers for biodosimetry,^164^, ^165^, ^166^ this area holds promise for more personalized treatment regimens in the future when we will hopefully have more drugs available and are more informed by biomarkers addressing biological responses and predicting future complications.^167^

Beyond the logistical and diagnostic challenges discussed above, emergency responders face additional hazards in the field. Critically, it is essential to recognize that in the context of an actual radiological or nuclear crisis, animals (both wildlife and companion animals) should be regarded as potential vectors of external radioactive contamination,^168^ which may pose a secondary exposure risk to responders, medical personnel, and the public during rescue or decontamination efforts. This operational reality necessitates specialized protocols for handling contaminated animals in the field.

### 6.3 Biological accessibility and applicability

The biological accessibility and population‐wide applicability of current and emerging radiation MCMs require critical consideration alongside their development. Although significant progress has been made, particularly for H‐ARS, the universal applicability of these solutions faces limitations. Not all individuals can readily undergo every proposed treatment due to factors such as specific contraindications, the need for human leukocyte antigen (HLA) matching in stem cell therapy, or undefined safety profiles in vulnerable populations, including children, pregnant individuals, and those with significant comorbidities. Furthermore, resource constraints during mass casualty incidents necessitate prioritization strategies, potentially favoring groups such as first responders and military personnel for pre‐exposure prophylaxis, or focusing immediate cytokine support on those exhibiting clear signs of H‐ARS. Addressing these limitations to universal accessibility and defining evidence‐based prioritization frameworks is a crucial aspect of comprehensive radiation emergency preparedness, which must be integrated into future research, development, and stockpiling planning efforts.

## 7 CONCLUSION

The current formulary for radiation emergency preparedness is limited, and more advanced efforts should be made, not only from the perspective of expanding the product portfolio to address gaps, but also with regard to guideline development, regulatory adaptability, and technology validation. Thus, a unified strategy, such as a research and development roadmap, is of vital importance, as it enables the entire process to be more focused, streamlined, and effective.

## AUTHOR CONTRIBUTIONS

**Wen‐Bing Ma:** Investigation; writing – original draft. **Rong‐Hua Yin:** Investigation; methodology; writing – original draft. **Guang‐Ming Ren:** Funding acquisition; writing – original draft. **Yin Zhang:** Writing – original draft. **Li‐Juan Li:** Writing – original draft. **Wei Liu:** Writing – original draft. **Ting Chen:** Writing – original draft. **Wei Zhou:** Writing – original draft. **Mian Zu:** Writing – original draft. **Rong‐Ling Yin:** Writing – original draft. **Xin‐Di Shi:** Writing – original draft. **Lei Wang:** Conceptualization; writing – review and editing. **Xiao‐Ming Yang:** Conceptualization; methodology; supervision; writing – review and editing.

## FUNDING INFORMATION

This study was supported by Beijing Nova Program (20220484086).

## CONFLICT OF INTEREST STATEMENT

The authors declare that they have no competing interests.

## ETHICS STATEMENT

Not applicable.

## CONSENT FOR PUBLICATION

Not applicable.
